# A Rare Case of Metastatic Carotid Body Paraganglioma: A 7-Year Asymptomatic Period

**DOI:** 10.1155/crom/5565079

**Published:** 2025-10-22

**Authors:** Vishal Parackal, Vibha S. P., Mukesh Shanthilal

**Affiliations:** ^1^Independent Investigator, Mysore, Karnataka, India; ^2^Community Medicine, SVYM Palliative Care Center, Mysore, Karnataka, India; ^3^Department of Oncology, Mysore Medical College and Research Institute, Mysore, Karnataka, India

## Abstract

Paragangliomas are rare neuroendocrine tumors that arise from chromaffin cells that can be sympathetic or parasympathetic in nature. Paragangliomas are closely related to pheochromocytomas, which are also a form of neuroendocrine tumor arising from the adrenal medulla (Chen et al., 2010; García-Carbonero et al., 2021). Paragangliomas of sympathetic origin are often secretory in nature, causing symptoms such as headache, palpitations, excessive perspiration, and high blood pressure, and are most often found along the sympathetic chain. Common locations include the abdomen, skull base, bladder, and aortic bifurcation. Paragangliomas found in the head and neck region are often parasympathetic in origin and are non-functional. The annual incidence of paragangliomas varies from 0.04 to 0.9 individuals per 100,000 population (Subhi et al., 2022) and can present in any age group. The average age of diagnosis ranges from the third to fifth decade based on the nature of the tumor (Eisenhofer et al., 2011), and there is no significant gender predilection. These tumors are often associated with germline mutations in VHL, RET, NF1, SDHA,MEN2, SDHB, SDHC, SDHD, SDHAF2 genes (Timmers et al., 2007; Lefebvre and Foulkes, 2014; Fliedner et al., 2010). Most cases of paragangliomas are benign with very low potential for metastasis; overall, paragangliomas have a 0-36% chance of metastasis (Fliedner et al., 2010; O'Riordain et al., 1996) often being sympathetic in origin. Metastasis is more common in patients with a germline mutation in SDHB gene and having a primary tumor size greater than 5 cm at presentation (Araujo-Castro et al., 2023; Lam, 2017). This case highlights an unusual clinical course: a carotid body paraganglioma, initially asymptomatic and successfully resected, developed skeletal metastasis after a prolonged disease-free interval of 7 years. This report underscores the importance of revisiting conventional risk stratification, incorporating genetic testing, and ensuring vigilant, long-term follow-up.

## 1. Introduction

Paragangliomas are rare neuroendocrine tumors that arise from chromaffin cells that can be sympathetic or parasympathetic in nature. Paragangliomas are closely related to pheochromocytomas, which are also a form of neuroendocrine tumor arising from the adrenal medulla [1, 2]. Paragangliomas of sympathetic origin are often secretory in nature, causing symptoms such as headache, palpitations, excessive perspiration, and high blood pressure, and are most often found along the sympathetic chain. Common locations include the abdomen, skull base, bladder, and aortic bifurcation. Paragangliomas found in the head and neck region are often parasympathetic in origin and are nonfunctional. The annual incidence of paragangliomas varies from 0.04 to 0.9 individuals per 100,000 population [3] and can present in any age group. The average age of diagnosis ranges from the third to fifth decade based on the nature of the tumor [4], and there is no significant gender predilection. These tumors are often associated with germline mutations in VHL, RET, NF1, SDHA, MEN2, SDHB, SDHC, SDHD, and SDHAF2 genes [5–7]. Most cases of paragangliomas are benign with very low potential for metastasis; overall, paragangliomas have a 0%–36% chance of metastasis [7, 8] often being sympathetic in origin. Metastasis is more common in patients with a germline mutation in the SDHB gene and having a primary tumor size greater than 5 cm at presentation [9, 10]. This case highlights an unusual clinical course: a carotid body paraganglioma, initially asymptomatic and successfully resected, developed skeletal metastasis after a prolonged disease-free interval of 7 years. This report underscores the importance of revisiting conventional risk stratification, incorporating genetic testing, and ensuring vigilant, long-term follow-up.

## 2. Case Report

**Figure 1:**
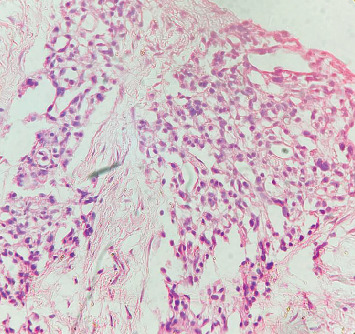
H & E section of the biopsy from the chest wall.

**Figure 2:**
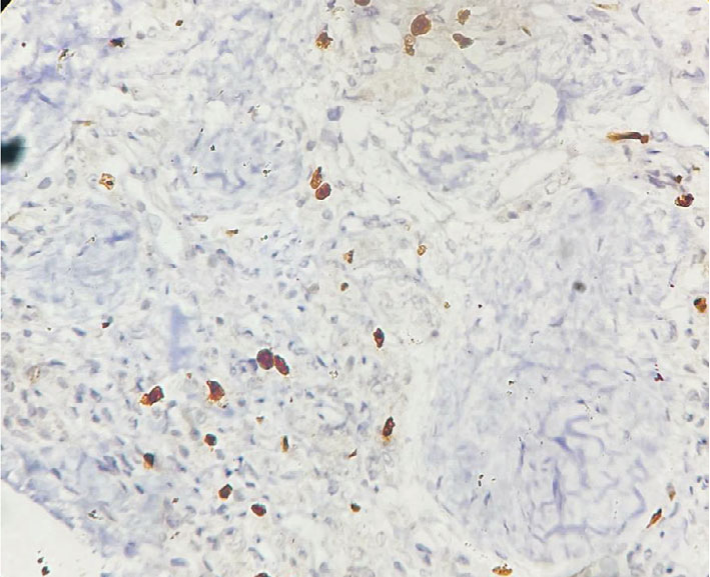
Immunohistochemistry-chromogranin positive cells.

**Figure 3:**
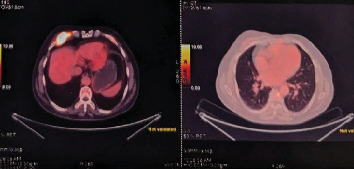
PET CT postchemotherapy showing the chest wall invasion.

The patient, aged 54 years in 2013, first noticed a small mass on the right side of the neck that was asymptomatic. The mass was not painful, was slow-growing in nature, and did not cause any compressive symptoms, causing the patient to ignore the mass. In 2017, the patient started experiencing discomfort in his neck due to the mass and had noticed significant growth of the same. MR angiography done showed the presence of an irregularly mixed signal intensity heterogeneously enhancing soft tissue lesion within the carotid sheath on the right side of the carotid bifurcation level, with splaying of the carotid arteries with multiple feeding vessels arising from the right ECA, and no evidence of invasion into the adjacent structures. The patient underwent resection of the tumor measuring 5 × 4 × 3 cm. The histopathological report of the specimen showed a circumscribed tumor composed of round to oval cells with granular cytoplasm and vesicular nuclei. The tumor cells were arranged in nests with a zellballen appearance. This confirmed that the carotid body tumor resected was a paraganglioma. A detailed history and conversation with the patient confirmed that there was no family history of similar tumors. The patient's postoperative period had no complications, and the patient remained symptom-free. Seven years postsurgery, the patient started experiencing a sense of chest tightness and discomfort in his upper back. He noticed a mass on his chest wall. The patient had no symptoms suggestive of catecholamine excess, such as palpitations, episodic hypertension, diaphoresis, or headaches. Examination of the patient showed the presence of an irregular, hard mass on the right side of the anterior chest wall lateral to the sternum. An 18F-FDG PET-CT scan performed on 20/1/24 revealed the presence of multiple active skeletal lesions of suspected neoplastic etiology, with no evidence of active lesions in the previous surgical site and no evidence of a second primary tumor in the adrenals or along the sympathetic chain. CT-guided biopsy of the mass showed the presence of fibrocollagenous tissue with multiple nests of polyhedral cells with granular cytoplasm, vesicular nuclei, dispersed chromatin surrounded by fibrovascular tissue suggestive of paraganglioma deposits (Figure 1) with a GAPP score of 4 indicating a moderately differentiated tumor. The tumor was positive for immunohistochemical markers synaptophysin and chromogranin and negative for PanCK (Figure 2). The patient was started on chemotherapy with carboplatin, etoposide, atezolizumab, and underwent five cycles of the same along with zolendronic acid and octreotide. Repeat 18F-FDG PET-CT done following chemotherapy showed a few new foci of skeletal lesions and a mild interval increase in the soft tissue component of a few lesions (Figures 3). Ga68 DOTANOC was done at the same time, noting accumulation in the region of the skeletal lesions, SUV: 86.43.

## 3. Discussion

This case challenges the traditional understanding of carotid body paragangliomas, which are often considered low risk for metastasis due to their parasympathetic origin and nonfunctional behavior [11]. The patient under discussion was initially asymptomatic and presented only with complaints of a mass on the right side of the neck and discomfort associated with the same. Although symptoms emerged 7 years postresection, the metastatic lesions likely predated clinical detection, highlighting the importance of earlier functional imaging and long-term surveillance. The 10% rule, as described for paragangliomas and pheochromocytomas, no longer holds true, as newer diagnostic modalities and advancements in genetic studies have shown that the metastatic potential for paragangliomas ranges from 0% to 36% [7, 9]. The highest predictor for metastasis remains mutation in the SDHB gene; other factors implicated include the size of the tumor at the time of presentation, location of the primary tumor, with glomus jugular tumors having higher chances of metastasis when compared with carotid body tumors, and the type of tumor, with sympathetic tumors having a higher chance of metastasis [12]. The diagnosis of a malignant paraganglioma requires the presence of metastasis to sites that are usually devoid of enterochromaffin cells [1], thereby making functional imaging modalities, such as PET-CT, an important screening tool for detecting metastasis in an otherwise healthy individual. Established risk factors for metastasis, such as tumor size ≥ 5 cm and genetic mutations, particularly in SDHB [5, 7], were likely contributors to this outcome. However, genetic testing was not performed initially, highlighting a gap in standard diagnostic protocols. This case reinforces the necessity of extended postresection imaging surveillance and consideration of genetic testing in all patients with paragangliomas, even when initial prognostic indicators suggest low risk.

## 4. Conclusion

This case highlights the atypical presentation of metastatic paraganglioma, underscoring the importance of continuous long-term follow-up in patients with carotid body paragangliomas, regardless of initial risk stratification. The delayed metastatic course over 7 years challenges conventional predictors and highlights the need for integrating genetic screening and updated surveillance protocols to enable early detection of recurrence. For similar cases, we recommend baseline whole-body functional imaging at diagnosis, genetic testing (including SDHB immunohistochemistry when sequencing is unavailable), and biochemical screening. Postresection surveillance should include cross-sectional and/or functional imaging annually for at least 10 years and lifelong follow-up in genetically positive or high-risk patients to enable early detection of recurrence or metastasis. In patients with confirmed SDHB mutations, annual plasma-free metanephrine levels are recommended, irrespective of symptoms, given the ongoing risk of second primaries and biochemical recurrence.

## Data Availability

No datasets were generated or analyzed during the current study. All relevant clinical details are included within the article.
